# Impact of the coronavirus pandemic (COVID-19) on the professional practice and personal well-being of community pharmacy teams in the UK

**DOI:** 10.1093/ijpp/riab062

**Published:** 2021-10-04

**Authors:** Sukvinder Kaur Bhamra, Jasmin Parmar, Michael Heinrich

**Affiliations:** 1 Medway School of Pharmacy, University of Kent, Chatham, Kent, UK; 2 UCL School of Pharmacy, London, UK

**Keywords:** coronavirus, COVID-19, pandemic, pharmacy, wellbeing

## Abstract

**Objectives:**

Community pharmacy teams (CPTs) were at the frontline of dealing with patients throughout the COVID-19 pandemic. This study aimed to explore the impact on professional practice and personal well-being of CPTs, in the UK.

**Methods:**

A 25-item survey was designed including a range of open and closed questions. The survey was piloted before being published online via SurveyMonkey and distributed using social media platforms. A combination of opportunity and snowball sampling was employed to recruit participants who worked in community pharmacy (CP) during the pandemic.

**Results:**

In total 758 participants (75% completion rate) including pharmacists, owners, managers, technicians, dispensers, healthcare assistants and pre-registration pharmacists took part. Increased workloads and working hours coupled with staff shortages compromised professional practice (*n* = 257, 35%). Some of the key challenges of working in CP during the pandemic included: a fear of contracting and passing the virus to others (*n* = 578, 78%), patients stockpiling medicines (*n* = 530, 71%) and doctors’ surgeries being closed (*n* = 517, 70%) The impact on emotional well-being (*n* = 433, 76%) included stress, anxiety, depression and loneliness; physically (*n* = 322, 56%) it affected sleep, pain and weight. The effects of the pandemic left 45% (*n* = 258/569) of participants reconsidering their future in CP as they felt demotivated, unsupported and undervalued.

**Conclusion:**

Despite the enhanced pressures and lack of initial recognition CPTs played a vital role in caring for the population during the pandemic. Resources to better support pharmacy teams in the future not only rely on more funding for better provisions but also investing in CPTs’ well-being.

## Introduction

On 11 March 2020, the World Health Organisation (WHO) declared the coronavirus pandemic (COVID-19) as a global health crisis, at which point more than 118 000 cases had been reported in 114 countries.^[1]^ Since then, there has been a high level of infections with countries like the USA, Mexico, India, Brazil and the UK having been affected particularly badly.^[2]^ The initiation of national lockdowns, social distancing measures and quarantine protocols to prevent the spread of the virus has had widespread implications on healthcare systems around the world.^[3]^

In the UK, in the first 6 months (1 February–31 July 2020) 313, 483 positive COVID-19 cases were identified with over 41 000 deaths. In the second 6 months (1 August 2020–31 January 2021) over 3.8 million cases and 109 000 deaths were recorded.^[4]^ The National Health Service (NHS) was overwhelmed with cases of COVID-19 leading to the creation of temporary hospitals and military support for the NHS.^[5]^

At the start of the pandemic doctors’ surgeries closed their doors to patients as general practitioners (GPs) switched to digital consultations.^[6]^ This left community pharmacy teams (CPTs) at the frontline to deal with patients face-to-face. Pre-registration pharmacists (pre-regs) were unable to sit the 2020 General Pharmaceutical Council (GPhC) registration assessment and provisional registration was introduced to enable them to practice as a pharmacist under certain conditions (i.e. under the guidance of a senior pharmacist; not working as a locum).^[7]^ Despite being the first port of call for patients, pharmacists and their teams were not explicitly defined as key workers in the list of key workers published on 18 March 2020.^[8]^ This led to considerable frustration and backlash from pharmacy professional representative bodies which led to the then Health Secretary for England, Matt Hancock, making a statement identifying community pharmacists as critical key workers and thanking them for their contribution.^[8, 9]^

The public anxieties in the initial phase of the pandemic led to patients stockpiling prescription and over-the-counter (OTC) medicines. Consequently, temporary shortages and some price increases were seen.^[10]^ The interest in adjuvant and complementary therapies for the prevention and treatment of COVID-19 was promoted through social media.^[11]^

During this first phase various surveys were launched, to explore the immediate effects of the pandemic on pharmacists. The Pharmacy Magazine commissioned a series of surveys that identified key challenges such as lack of personal protective equipment (PPE) provided to pharmacists, stock shortages, rising costs of medicines, staff absences and increased workloads.^[12, 13]^ The information presented remains superficial, lacking depth and personal perspectives. There has been little systematic research into the effects of the pandemic on personal well-being and mental health of CPTs and most focused solely on pharmacists.^[14]^

The overarching aim of this study was to explore the impact of the coronavirus (COVID-19) pandemic on UK-based CPTs’ professional practice and personal well-being to plan for future crises.

Objectives of the study were to:

- Identify the challenges CPTs faced during the COVID-19 pandemic which impacted their professional practice.
- Evaluate the impact COVID-19 had on the personal well-being of CPTs.
- Explore patients’ use of OTC medicines and complementary therapies to prevent, manage and treat the symptoms of COVID-19.
- Review the effect of the delayed registration assessment for pre-registration pharmacists; looking at the impact on pre-registration pharmacists during the pandemic and the effect on their career progression.

## Methods

### Questionnaire design

A 25-item self-completion questionnaire was designed to be distributed online using SurveyMonkey. A range of open and closed questions and scaled responses were used. The questionnaire was piloted (*n* = 8) and re-drafted with a subsequent pilot (*n* = 6), with a sub-sample of the final population, who were recruited from personal CP networks.

The final questionnaire (Supplementary Material 1) was subdivided into six sections including: impact on professional practice, resources and information, patient experience, complementary and adjuvant therapies, well-being and demographics. There was an additional section specifically for pre-registration pharmacists to consider the impact of the delayed registration assessment. No personal data was collected, so responses were anonymous. A participant information leaflet was included at the start of the online survey. Participants were asked to read this and then formally consent. An initial screening question confirmed that they had worked in CP during the pandemic. If consent and CP job role were not confirmed, the respondents were disqualified from continuing the survey. Participants were offered the chance to enter an optional prize draw, of two £50 gift vouchers, as an incentive for completing the questionnaire.

### Ethics

Ethical approval was obtained from the Medway School of Pharmacy Research Ethics Committee (REF010420) before commencing.

### Participant recruitment and survey administration

A combination of opportunistic and snowball sampling was employed. The link for the online survey was shared by the research team via personal and professional networks and using social media (i.e. LinkedIn and Twitter). Posts were refreshed weekly to increase visibility of the survey. As the link was re-shared by participants it led to snowball sampling. Settings within the survey software were enabled to prevent multiple attempts from the same respondent. The questionnaire was available from 15 June to 31 October 2020, during the first phase of the pandemic, thus the data does not reflect changes in opinions over time.

The inclusion criteria required participants to be over 18 years of age, having worked in CP, in the UK, during the pandemic while the survey was open. The survey was inclusive of all members of CPTs, including pre-regs, pharmacists, pharmacy technicians, accredited checking technicians (ACT), dispensers, healthcare assistants (HCA), managers, owners and other members of the pharmacy team. Those working in other pharmacy settings such as hospital, industry and academia were excluded from participation.

### Data analysis

The Statistical Package for the Social Sciences software (V.27) was used for the analysis of closed questions and statistical analysis (i.e. descriptive frequencies and chi-squared tests). Microsoft Excel was used to create the graphical representations. NVivo was used for content and thematic analysis of open-ended questions. Thematic analysis involved coding responses to identify emerging themes.^[15]^ An identification number was assigned to each completed form (e.g. P001).

## Results

A total of 986 respondents started the questionnaire, 39 were disqualified because they did not provide consent or they did not meet the eligibility criteria, and 189 did not complete any of the questions. Thus, 758 evaluable questionnaires were included in the final sample for analysis. Question completion rate decreased with progression through the survey as indicated in the reported results.

### Participant demographics

Table 1 summarises the demographic data of participants. A large proportion of participants were female (*n* = 384/560, 69%), in the 21–30 years age group (*n* = 261, 47%) and White-British (*n* = 258, 46%) (Table 1).

**Table 1:** Participants’ demographic data

		Number of participants (*n*)	Proportion of sample (%)
Gender(*n* = 560)	Female	384	69
	Male	170	30
	Non-binary	3	0.5
	Prefer not to say	3	0.5
Age(*n* = 560)	<20	12	2
	21–30	261	47
	31–40	147	26
	41–50	73	13
	51–60	55	10
	61+	12	2
Ethnicity(*n* = 560)	White: British	258	46
	White: Other	42	7
	Black/Black British: African/Caribbean	32	5
	Black/Black British: Other	2	0.4
	Asian/Asian British: Indian/Pakistani/Bangladeshi/Sri Lankan	171	31
	Asian/Asian British: Chinese	8	1
	Asian/Asian British: Other	26	5
	Other	21	4
Job role(*n* = 758)	Accredited checking technician	38	5
	Delivery driver	1	0.1
	Dispenser	159	21
	Healthcare assistant	24	3
	Manager	15	2
	Pharmacist	365	48
	Pharmacy advisor	1	0.1
	Pharmacy owner	2	0.3
	Pharmacy undergraduate student	6	0.8
	Pre-registration pharmacist	79	10
	Provisional pharmacist	1	0.1
	Technician	67	9
Years of practice in pharmacy(*n* = 546)	Pre-registration	83	15
	1–5	192	35
	6–10	93	17
	11–20	89	16
	21–30	56	10
	31+	33	6
Type of community pharmacy worked for(*n* = 557)	Independent	169	30
	Chain	364	65
	Combination of independent and chain	24	4
Country of residence(*n* = 557)	England	497	89
	Northern Ireland	7	1
	Scotland	38	7
	Wales	15	3

### Access to resources and information sources

**Figure 1:**
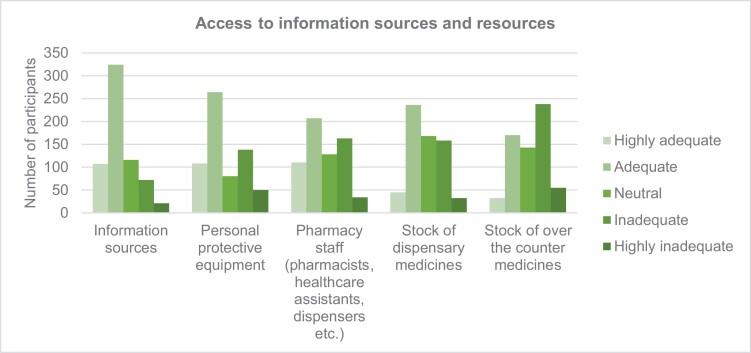
Adequacy of access to information sources and resources for community pharmacy teams during the COVID-19 pandemic.

Participants (*n* = 644) ranked the adequacy of information sources and availability of resources on a scale from highly adequate to highly inadequate (Figure 1). The quantitative data identified information sources, PPE, staffing and stock of dispensary medicines as highly adequate/adequate throughout the pandemic. While the supply of OTC medicines was commonly ranked as inadequate/highly inadequate. However open text responses revealed a more nuanced experience as shown below.

### Personal protective equipment

Many participants (*n* = 102) commented on how, at the start of the pandemic, PPE was not easily accessible but availability improved overtime.

**P447:** For several weeks, PPE was not available whatsoever and GPs closed their doors but how could we? We just got both pharmacy and GP workload thrust onto us and it was very isolating. This was further emphasised when we weren’t provided any protection but had to just deal with the increase in demand. (Pharmacist)

In most cases (*n* = 579/638, 91%) employers provided PPE; while some pharmacies received PPE from the NHS (*n* = 153, 24%). References of having to source PPE personally (*n* = 172, 27%), from colleagues (*n* = 96, 15%), from patients (*n* = 53, 8%) and alternative sources (*n* = 49, 7%) such as donations from local schools/colleges and community volunteers were also noted.

**P319:** Not enough PPE was available during the peak of the pandemic, and was reliant on donations from patients and other shops. (Dispenser)

### Changes to supply and demand of medication

The demand to fulfil prescriptions initially increased as patients tried to request medicines more frequently and in higher volumes. However, stock of most prescription medicines was deemed to be adequate and supplied sufficiently by wholesalers and pharmacies respectively. Participants (*n* = 124) noted the medicines which were in high demand and became difficult to obtain included inhalers and analgesics (i.e. codeine, paracetamol and ibuprofen). The increased demand subsequently affected the purchase price (from wholesalers) and sale price (to patients). Stock of OTC medicines was ranked as being inadequate which was reinforced by comments (*n* = 54) related to increased demand, patients stockpiling and panic buying:

**P519:** For some months, there was a national shortage of paracetamol tablets and capsules over the counter due to the increase in demand. Additionally, paracetamol and ibuprofen oral solution for children was in short supply. These products were on a quota which meant the pharmacy could only buy a limited quantity per month. In a day, it was not unusual for 50 boxes (16 tablets in a box) of paracetamol tablets to be sold (over the counter). One box per person. (ACT)

### Impact on community pharmacy teams’ professional practice

The vast majority of participants (*n* = 704/746, 94%) reported an increased workload, which was independent of pharmacy type (chain or independent pharmacy) (χ ^2^ = 1.94, df = 4, *P* = 0.746). Working hours were increased for 59% (*n* = 442/743) of participants, beyond normal opening hours. Some participants said there was no change in workload (*n* = 11, 1.5%) or working hours (*n* = 234, 31%) because their pharmacies had fixed opening hours. A small proportion claimed their workload (*n* = 31, 4%) and working hours (*n* = 67, 9%) decreased due to staff shortages and reduced footfall. There were 300 open text responses illustrating the impact on professional practice (Table 2; Supplementary Material 2).

**Table 2:** Key factors which impacted professional practice of community pharmacy teams

Core themes	Participant quotes
Communication	**P333:** ‘The implementation of social distancing guidelines and the use of face masks make it harder to understand patients and to communicate effectively. I have seen instances where hand-out errors happen’ (Pharmacist)
Clinical appropriateness	**P647:** ‘This was a new community pharmacy I was practicing at, with little guidance on the first Saturday after lockdown restrictions were lifted. Heavy footfall and little support. Many errors with prescriptions and many emergency supply requests as GP practices were shut. Clinical judgement had to be exercised more so than ever. Assessing requests for emergency supply was difficult and often stressful’ (Pharmacist)
Increased workload	**P505:** ‘Workload tripled in days with less staffing due to COVID related sickness/isolation in the beginning. Working to bring in measures to stay safe for both patients and colleagues alike without any official guidelines and then to adhere to guidelines. Extra pressure due to GPs telling patients to go to pharmacies for example for a blood pressure check as they were unable to see them face to face’ (Pre-registration Pharmacist)
Medication requirements	**P736:** ‘People were stockpiling medicines so workload went up very quickly. Hard to communicate through masks which increased the risk of making handout errors’ (Pharmacist)
Near misses/mistakes	**P631:** ‘The main impact the pharmacy noticed was an increase of dispensing errors that fortunately, didn’t leave the pharmacy. We tried moving to different jobs in the pharmacy which helped temporarily but the team fatigued just as quickly’ (Technician)
Provision of services	**P753:** ‘The increased workload meant that other services, non-essential and advanced and any patient advice that needed to be given was cut back. Prescriptions ended up rolling into the next days work which put us back’ (Pharmacy student)
Staff shortages	**P040:** ‘Huge impact as lost experienced staff to shielding and had to train new staff. Financial implications of changes to staff and way we work. Stress due to lack of information on best practice. Had to “wing it” and hope for the best’ (Dispenser)
Standard operating procedures (SOPs)	**P105:** ‘Disregarding SOPs or making short cuts in attempt to deal with the workload. At times had to authorise sale of OTC medication beyond their licensing so as to help the patient who was unable to see a prescribing clinician. Working hours luckily did not change as the team decided to be strict on themselves in regards to hours worked to reduce risk of exposure however the increased workload would normally have warranted some extension of the working hours’ (Technician)
Working hours	**P436:** ‘At the start of the pandemic our workload increased by unbelievable amounts then after 2 weeks it went really quiet as most students went home. In our situation pharmacy staff not being allowed to be furloughed was a struggle as we were very quiet’ (Dispenser)

Participants were asked if they had experienced any of a list of potential challenges, identified throughout the literature. Responses included: a fear of passing the virus to people they lived with (*n* = 578/743, 78%), patients stockpiling medicines (*n* = 530, 71%), GP surgeries being closed (*n* = 517, 70%), coping with family while working (*n* = 296, 40%), compromised professional practice (*n* = 257, 35%), contracting the virus (*n* = 225, 30%) and increased cost of medicines (*n* = 23, 31%). Table 3 summarises the themes identified, in the open text responses (*n* = 415), of the key challenges encountered by participants.

**Table 3:** Challenges encountered by community pharmacy teams while working through the pandemic

Key themes	Challenges encountered
Medication	• Increased demand and limited supply of medicines• Increased wholesaler price impacted price of OTC medicines• Unlicensed supply of medicines as GPs and dentists were unavailable
Patients	• Attitudes – abusive, frustrated and worried patients• Expectations exceeded capabilities of what pharmacies could provide• Experience – reduced services and increased waiting times
Professionalism	• Clinical decisions made outside of normal practice as other HCPs were not accessible• Compromised professional practice as SOPs were not always followed• Restricted ability to continue learning/development
Safety	• Controlled entry of patients to enable social distancing• Enhanced cleaning and enforcement of PPE – to maintain patient and staff safety• Shielding of vulnerable staff – shortages led to increased pressure on working staff
Workload	• Increased workload and working hours• Enhanced pressure impacted well-being• Services limited as backlog of work piled up

### The delayed registration assessment

**Figure 2:**
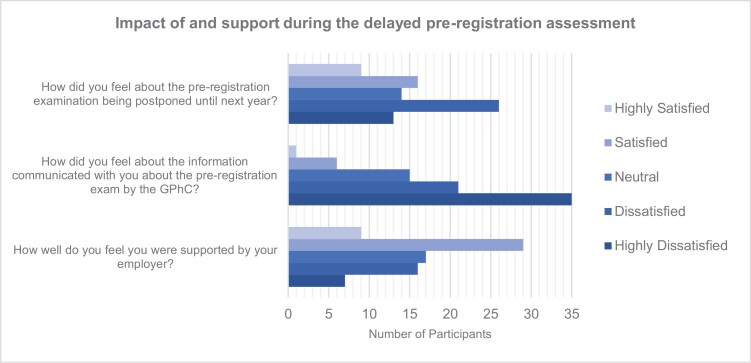
Impact of the delayed pre-registration assessment and support from the GPhC and employers.

Of the total sample, 10% (*n* = 79) identified as being pre-registration pharmacists. All but one of the pre-regs continued to work in CP during the pandemic. There were mixed feelings about the delayed registration assessment but a clear dissatisfaction with the communications from the GPhC (Figure 2).

Some pre-regs (*n* = 25, 32%) were happy with the delayed assessment as the pandemic was an unnerving time which created additional pressures and anxieties:

**P353:** I felt it was a good idea to delay the exam however it should have been cancelled completely due to such a long delay with an uncertain future since pre reg trainees have demonstrated true pharmacist skills during the pandemic. (Pre-reg)

Others (*n* = 14, 18%) had mixed feelings as they believed it gave them more opportunities to learn while working through the pandemic and more time to revise for the assessment however, they were conscious of the impact on their future careers. While half of pre-regs (*n* = 39, 50%) expressed their fears and frustration as they were prepared to sit the exam:

**P555:** I was fully prepared to sit my pre-registration exam despite the very challenging time we faced. There was a severe lack of communication between the GPhC and the pre-registration trainees and it often felt that we had just been left to one side. (Pre-reg)

The Royal Pharmaceutical Society (RPS) was identified as the most helpful (*n* = 38) source of support and information about the postponed registration assessment; followed by the Pharmacists’ Defence Association (PDA) (*n* = 34), employers (*n* = 23), the GPhC (*n* = 15) and other sources (*n* = 10) including universities and training providers.

The impact of the delayed assessment affected future career plans of 71% (*n* = 55/77) of pre-regs, that is, preventing them from qualifying as pharmacists, starting their professional careers or continuing with further education. Thirteen pre-regs (*n* = 13/78) stated they were hoping to continue with further education to undertake: a clinical diploma, prescribing and management course, Masters, Medicine degree or a PhD, which was delayed as a result of not qualifying in the expected timeframe.

### Impact on personal well-being of community pharmacy teams

Well-being was considered emotionally and physically; 76% (*n* = 433/569) of participants reported how their emotional well-being had been affected resulting in feeling: stressed (*n* = 137), anxious (*n* = 74), worried (*n* = 24), scared (*n* = 15), depressed (*n* = 14) and lonely (*n* = 8). Effects on physical well-being were reported by 56% (*n* = 322/571) of participants including feeling tired (*n* = 95) and exhausted (*n* = 50), and experiencing: insomnia (*n* = 20), weight fluctuations (*n* = 28), headaches/migraines (*n* = 11), pain (*n* = 6) and hair loss (*n* = 5).

A list of expressions were provided for participants (*n* = 571) to select how they had felt. The main positive feelings reported included: empowered (*n* = 105), determined (*n* = 187), motivated (*n* = 167), proud (*n* = 262) and resilient (*n* = 252); additional positive feelings reported by smaller numbers included caring, empathetic, hopeful, lucky and thankful. Negative phrases included anxious (*n* = 277), frustrated (*n* = 291), demotivated (*n* = 158), discouraged (*n* = 138) and resentment (*n* = 118); with smaller numbers reporting feeling angry, disappointed, irritated, lonely, overwhelmed, unappreciated, unsupported and undervalued.

Participants expressed mixed emotions of working in CP during the pandemic:

**P306:** Proud to work and help in the crisis. Demotivated and disheartened by the attitudes to pharmacy workers. Anxious about catching COVID-19 and fear of assault by customers. (Dispenser)

**P499:** We felt everything. Overall, it drove home what I have felt for a while. As pharmacists we are underpaid, undervalued and not as well respected as we should be. (Pharmacist)

**P690:** Wish there was more support for pharmacists to help with emotional trauma, stress, to become more resilient. We think about everyone but ourselves and that can really put a person in a very sad place. (Pharmacist)

The increased pressure and stress led 45% (*n* = 258/569) of participants to reconsider their career in CP. There was a statistically significant association between years of experience working in CP and reconsidering a career in CP (χ ^2^ = 19.6, df = 5, *P* = 0.002). Participants with longer service in CP were less likely to reconsider their careers in CP compared with those at earlier stages of their careers. Some participants commented that they had already left or handed in their notice, and how this was the ‘final straw’. A similar number of participants (*n* = 234/569, 41%) were determined to continue with their careers in CP as they acknowledged their passion for their profession.

### Patients’ attitude and expectations of community pharmacy teams

Participants described an assortment of positive and negative changes in their patients’ characteristics and behaviours towards them during the pandemic (Table 4; Supplementary Material 3).

**Table 4:** Changes in patients’ attitudes and expectations towards community pharmacy teams

Patients’ attitudes and expectations	Key terms identified (*n*)	Participant quotes
Positiveattributes	Kind (*n* = 11)	**P527: ‘**General kindness shown by patients coming in bringing food if we didn’t have time for lunch breaks and asking if we were ok or needed anything’ (Dispenser)
	Grateful (*n* = 18)	**P010:** ‘I felt there was more patience seen with customers when prescriptions weren’t ready. Patients were more thankful and grateful with the service we provided even though this is what we have always done. Covid as made patients realise how hard we actually work’ (Pharmacist)
	Understanding (*n* = 24)	**P757:** ‘As a whole people were lovely and really understanding. They were thankful that we were there and we were open. That we could give help and support and advice. A few in the panic of everything were very rude, scared and demanding. They wanted us to be their doctors, their pharmacists, their dentist, their delivery person. They wanted everything and expected it that second. It was a struggle when basics were unavailable e.g. paracetamol… Everyone expected us to have all the answers, and we didn’t. We were scared ourselves’ (ACT)
Negative attributes	Abusive (*n* = 36)	**P529:** ‘At the start of the lockdown they were in panic mode and were very demanding and at times verbally abusive if they didn’t get what they want but the attitudes did change a lot as time went on and we got positive feedback from patients saying how helpful we have been during this time’ (Pharmacist)
	Aggressive (*n* = 29)	**P703:** ‘People were extremely aggressive on the verge of being violent’ (Pharmacist)
	Angry (*n* = 10)	**P306:** ‘Higher expectations, wanting prescriptions instantly and angry about having to wait or queue to enter the pharmacy. Unable to understand the massively increased workload and the stock shortages caused by all the extra prescriptions. Refusal to comply with social distancing requests. Constantly phoning as they couldn’t contact the doctors’ (Dispenser)
	Demanding (*n* = 50)	**P066:** ‘More demanding, more impatient, more aggressive (I was spat at when advised we didn’t have an item after patient had queued to get in store), more frustrated at not being able to see GP but not happy about what we could offer either’ (Pharmacist)
	Impatient (*n* = 23)	**P336:** ‘Patients were very frustrated and impatient in relation to the current climate and pandemic, it was felt that they took this out on the pharmacy staff’ (Dispenser)
	Rude (*n* = 35)	**P240:** ‘Aggressive and rude customer every day. We were sworn at due to the increased waiting time’ (Pharmacist)
	Selfish (*n* = 16)	**P732:** ‘More demand for quick turnarounds, more selfishness, more excuses for not putting prescriptions requests in on time, a higher demand for delivery, and more hostility towards the pharmacy and the GP surgeries’ (Healthcare assistant)

### OTC requests including supplements and complementary medicines

For the treatment and prevention of COVID-19 respondents (*n* = 246/585, 42%) reported that patients commonly enquired about echinacea garlic, ginger, lemon, turmeric and multivitamins/supplements, in particular, vitamins C and D. There were fewer enquiries relevant to the acute (*n* = 92/579, 16%) and recovery phase (*n* = 68/580, 12%). Participants indicated patients were becoming more proactive in managing their health and expressed a willingness to try alternative therapies to prevent contracting the virus.

Other OTC products which were in significantly higher demand during the pandemic, besides paracetamol, included codeine-based products (*n* = 264) and vitamins (*n* = 488). The sale of ephedrine-based products, echinacea, essential oils (e.g. menthol and eucalyptus), herbal remedies for anxiety and sleeping aids also saw some increases. Participants reported patients’ stocking up on these medicines ‘just in case’.

### Preparing for a future pandemic

**Figure 3:**
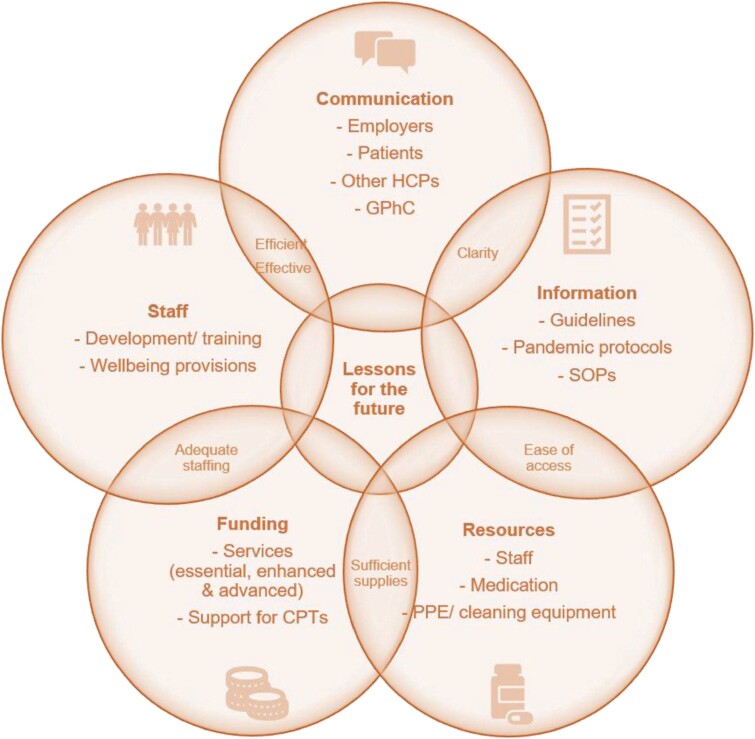
Key themes identified to help community pharmacies better prepare for a future pandemic.

Mixed views were expressed on how well-prepared community pharmacies were prepared to deal with the COVID-19 pandemic (Table 5; Supplementary Material 4). Participants were asked how community pharmacies could better prepare for a future pandemic (*n* = 435). Responses were coded into five major themes: communication, information, resources, funding and staff with several emerging subthemes contributing to lessons to be learned for the future (Figure 3).

**Table 5:** How community pharmacy teams felt they were prepared to deal with the COVID-19 pandemic

Preparedness for the pandemic	*n* (%)	Participant quotes
Well prepared	106 (17%)	**P031:** ‘We got ahead of the curve our staff agreed to isolate within their families to protect the team. I am extremely fortunate to have such a great team and with team work and local links feel we supported the staff and community’ (Pharmacist)
Not well prepared	247 (38%)	**P491:** ‘No, but we adapted very quickly- within 24 hours of receiving new information we had adapted’ (Pharmacist)
Somewhat prepared	264 (42%)	**P519: ‘**The nature of the pandemic made it difficult for the NHS to prepare for or have the resources to overcome the sudden increase in cases during the initial phase’ (Pre-reg)
Unsure	18 (3%)	**P733:** ‘More so than others’ (Healthcare assistant)

## Discussion

This study identified some of the key challenges which impacted CPTs’ professional practice and personal well-being during the COVID-19 pandemic in 2020. Despite the increased workload and working hours coupled with limited resources and staff, CPTs remained resilient and delivered an enhanced service, when other healthcare professionals were inaccessible. Suggestions for improving the future of community pharmacies to meet demands in such times rely on a combination of enhanced funding, better communication, access to resources, unified guidance and provisions for staff welfare.

The broad inclusion of all members of CPTs was unique to this study as it enabled perspectives of everyone in CP to be considered, rather than one particular role.^[12–14]^ The progressive question completion dropout rate could be attributed to the length of the survey, which could have been streamlined. The third question of the survey was open-ended, after which 99 participants dropped out, fewer open-ended questions could have been reserved towards the end of the questionnaire. Nevertheless, combining open and closed questions in this study was highly valuable as the level of detail participants disclosed may not have otherwise been achieved on such a large scale.

One of the key challenges identified by the majority of participants was the fear of contracting and passing the virus to others; globally, this has been a major concern for healthcare professionals.^[16–18]^ Hence, ensuring the safety of frontline workers was reportedly a national priority.^[19]^ Despite this, there were shortages in securing adequate supplies of PPE at the beginning of the pandemic.^[20]^ The difficulties some CPTs reported in sourcing PPE caused anger and frustration among participants who felt they were not considered as important as GPs who were provided with PPE but had closed their doors for face-to-face consultations.^[14, 21]^ Better communication from government, regulatory bodies, employers, other healthcare professionals, colleagues and patients was another core theme identified. For example, delayed and sparse communications from the GPhC led to confusion and misinformation, which left pre-regs feeling anxious about their future careers.^[22]^ Moreover, the implications of poor communication often left CPTs to resolve ethical dilemmas alone. Participants claimed they had to make decisions that were outside of their normal responsibilities, without consulting GPs. In September 2020, NHS England wrote to GPs to remind them to tell patients face-to-face consultations were available.^[6, 23]^ The anxiety and fear of the repercussions of the decisions CPTs made had a psychological impact on participants.^[14]^ To cope with the increased pressures and staff shortages some pharmacies were granted permission to reduce their opening hours or close for lunch^[24]^; but, this was not universal. Numerous accounts of verbal abuse were described; fortunately, no accounts of physical abuse were documented unlike a pharmacist from Southeast England who was assaulted by a patient.^[25]^

The uncertainty of the UK leaving the European Union triggered stockpiling of medicines^[26]^; which was further exacerbated during the pandemic and led the Department of Health and Social Care to urge suppliers to put contingency plans in place to prevent shortages.^[27]^ Guidelines for repackaging paracetamol from larger stocks to be sold OTC were introduced.^[28]^ The increased demand for medicines led to temporary shortages of some medicines (such as inhalers) and price increases.^[12, 13, 29]^ In addition to patients buying OTC medicines, such as analgesics, participants stated queries for complementary therapies for the prevention and treatment of the virus also increased as patients looked for alternative measures to manage their health.^[30]^ Participants identified their lack of knowledge of some of the remedies patients were enquiring about (i.e. concoctions of turmeric, ginger, honey and lemon) but more importantly the insufficient evidence to support the use of complementary therapies for COVID-19.

The All-Party Pharmacy Group (APPG) launched an inquiry to assess the impact of the pandemic on operational and financial pressures on pharmacies. Similar to this study, the APPG identified the increased workload CPTs faced and how they were having ‘to do more with less’.^[31]^ The APPG focused more on financial implications and provided some valuable recommendations for the future of pharmacy, highlighting that pharmacists should be commended for what they have done for the NHS and made suggestions for enhanced funding. The additional burden on CPTs affected well-being as participants reported feeling physically exhausted. A survey conducted by the RPS focused on mental health and well-being of pharmacists.^[14]^ Like this study, it also uncovered that the pandemic had a negative effect on mental health. Although the RPS survey was limited to pharmacists (inclusive of all sectors) the stresses and pressures identified were the same as those identified by participants in this study (i.e. long working hours, inadequate staffing and lack of support).

There were numerous accounts that highlighted the positive experiences of working in CP during the pandemic, such as patients being kinder and more considerate. General appreciation of CPTs was widely expressed by the public, government and various professional organisations.^[32, 33]^ The realisation of how valuable community pharmacies are may have taken some time but it did not go unnoticed.^[8, 9]^ However, the experience of working in CP during the pandemic led some participants to reconsidering their career in pharmacy. Similarly, the RPS survey uncovered a third of their participants (*n* = 316) had considered leaving the profession.^[14]^ Some of the reasons for leaving CP identified included a lack of recognition and support for the profession, salary, long working hours, feeling undervalued and unappreciated; these are similar to the reasons published by the GPhC for not renewing registration.^[34]^

Without the essential resources (such as medicines, PPE and information) required to provide services safely to the public, the challenges of working in CP during a pandemic were amplified. Suggestions for financial investments into CP were made by the APPG without which better preparation for the future would not be possible.^[31]^ Unified guidance and contingency plans which are communicated appropriately are vital to enable CPTs to work effectively. Ultimately plans to prevent reoccurrences of the events documented in this study need to be developed. Support for CPTs needs to be improved to enable them to continue practicing professionally but to also safeguard their personal well-being.

## Conclusion

The findings of this study have highlighted the pivotal role CPTs have had in providing frontline care for the UK population during the pandemic. CPTs remained accessible and a highly valuable resource, providing primary health care. More recognition needs to be given for their contribution. This study has provided a unique insight into how challenges encountered were managed and key lessons to take forward to better prepare for the future. Evidently, CP needs investment in resources, information and most importantly staff well-being. Overall, the preparedness of CPs as a community-based frontline service needs to be strengthened as a core response of the NHS in case of a pandemic.

## Supplementary Material

riab062_suppl_Supplementary_Table_2

riab062_suppl_Supplementary_Table_4

riab062_suppl_Supplementary_Table_5
