# Supplementary material for: Impact of the coronavirus pandemic (COVID-19) on the professional practice and personal well-being of community pharmacy teams in the UK
Source: Int J Pharm Pract. 2021 Oct 4:riab062. doi: 10.1093/ijpp/riab062 (PMC8500076; doi:10.1093/ijpp/riab062)
Supplement: riab062_suppl_Supplementary_Table_2 [file riab062_suppl_supplementary_table_2.docx]

Table 2 Key factors which impacted professional practice of community pharmacy teams

| *Core themes:* | *Participant quotes:* |
| --- | --- |
| Communication | **P333:** “*The implementation of social distancing guidelines and the use of face masks make it harder to understand patients and to communicate effectively. I have seen instances where hand-out errors happen.”* [Pharmacist] |
| Clinical appropriateness | **P647:** *“This was a new community pharmacy I was practicing at, with little guidance on the first Saturday after lockdown restrictions were lifted. Heavy footfall and little support. Many errors with prescriptions and many emergency supply requests as GP practices were shut. Clinical judgement had to be exercised more so than ever. Assessing requests for emergency supply was difficult and often stressful.”* [Pharmacist] |
| Increased workload | **P505**: “*Workload tripled in days with less staffing due to COVID related sickness/isolation in the beginning. Working to bring in measures to stay safe for both patients and colleagues alike without any official guidelines and then to adhere to guidelines. Extra pressure due to GPs telling patients to go to pharmacies for example for a blood pressure check as they were unable to see them face to face*.” [Pre-registration Pharmacist] |
| Medication requirements | **P736:** *“People were stockpiling medicines so workload went up very quickly. Hard to communicate through masks which increased the risk of making handout errors”*. [Pharmacist] |
| Near misses/ mistakes | **P631**: “*The main impact the pharmacy noticed was an increase of dispensing errors that fortunately, didn’t leave the pharmacy. We tried moving to different jobs in the pharmacy which helped temporarily but the team fatigued just as quickly*.” [Technician] |
| Provision of services | **P753:** *“The increased workload meant that other services, non-essential and advanced and any patient advice that needed to be given was cut back. Prescriptions ended up rolling into the next days work which put us back*.” [Pharmacy student] |
| Staff shortages | **P040:** *“Huge impact as lost experienced staff to shielding and had to train new staff. Financial implications of changes to staff and way we work. Stress due to lack of information on best practice. Had to ‘wing it’ and hope for the best.”* [Dispenser] |
| Standard operating procedures (SOPs) | **P105:** “*Disregarding SOPs or making short cuts in attempt to deal with the workload. At times had to authorise sale of OTC medication beyond their licensing so as to help the patient who was unable to see a prescribing clinician. Working hours luckily did not change as the team decided to be strict on themselves in regards to hours worked to reduce risk of exposure however the increased workload would normally have warranted some extension of the working hours*.” [Technician] |
| Working hours | **P436:** *“At the start of the pandemic our workload increased by unbelievable amounts then after 2 weeks it went really quiet as most students went home. In our situation pharmacy staff not being allowed to be furloughed was a struggle as we were very quiet.”* [Dispenser] |
