# Supplementary material for: Impact of the coronavirus pandemic (COVID-19) on the professional practice and personal well-being of community pharmacy teams in the UK
Source: Int J Pharm Pract. 2021 Oct 4:riab062. doi: 10.1093/ijpp/riab062 (PMC8500076; doi:10.1093/ijpp/riab062)
Supplement: riab062_suppl_Supplementary_Table_4 [file riab062_suppl_supplementary_table_4.docx]

*Table 4 Changes in patients’ attitudes and expectations towards community pharmacy teams*

| **Patients’ attitudes and expectations** | **Key terms identified (n):** | **Participant quotes:** |
| --- | --- | --- |
| **Positive**  **attributes** | **Kind**  (n=11) | **P527:** “*General kindness shown by patients coming in bringing food if we didn’t have time for lunch breaks and asking if we were ok or needed anything*”. [Dispenser] |
|  | **Grateful**  (n=18) | **P010:** “*I felt there was more patience seen with customers when prescriptions weren’t ready. Patients were more thankful and grateful with the service we provided even though this is what we have always done. Covid as made patients realise how hard we actually work*.” [Pharmacist] |
|  | **Understanding**  (n=24) | **P757:** *“As a whole people were lovely and really understanding. They were thankful that we were there and we were open. That we could give help and support and advice. A few in the panic of everything were very rude, scared and demanding. They wanted us to be their doctors, their pharmacists, their dentist, their delivery person. They wanted everything and expected it that second. It was a struggle when basics were unavailable e.g. paracetamol…Everyone expected us to have all the answers, and we didn’t. We were scared ourselves.”* [ACT] |
| **Negative attributes** | **Abusive**  (n=36) | **P529:** “*At the start of the lockdown they were in panic mode and were very demanding and at times verbally abusive if they didn’t get what they want but the attitudes did change a lot as time went on and we got positive feedback from patients saying how helpful we have been during this time*.” [Pharmacist] |
|  | **Aggressive**  (n=29) | **P703:**“ *People were extremely aggressive on the verge of being violent*.” [Pharmacist] |
|  | **Angry**  (n=10) | **P306:** “*Higher expectations, wanting prescriptions instantly and angry about having to wait or queue to enter the pharmacy. Unable to understand the massively increased workload and the stock shortages caused by all the extra prescriptions. Refusal to comply with social distancing requests. Constantly phoning as they couldn't contact the doctors*.” [Dispenser] |
|  | **Demanding**  (n=50) | **P066:** “*More demanding, more impatient, more aggressive (I was spat at when advised we didn’t have an item after patient had queued to get in store), more frustrated at not being able to see GP but not happy about what we could offer either*.” [Pharmacist] |
|  | **Impatient**  (n=23) | **P336:** “*Patients were very frustrated and impatient in relation to the current climate and pandemic, it was felt that they took this out on the pharmacy staff.”* [Dispenser] |
|  | **Rude**  (n=35) | **P240:** “*Aggressive and rude customer every day. We were sworn at due to the increased waiting time*.” [Pharmacist] |
|  | **Selfish**  (n=16) | **P732:** *“More demand for quick turnarounds, more selfishness, more excuses for not putting prescriptions requests in on time, a higher demand for delivery, and more hostility towards the pharmacy and the GP surgeries.”* [Healthcare assistant] |
