# Supplementary material for: Impact of the coronavirus pandemic (COVID-19) on the professional practice and personal well-being of community pharmacy teams in the UK
Source: Int J Pharm Pract. 2021 Oct 4:riab062. doi: 10.1093/ijpp/riab062 (PMC8500076; doi:10.1093/ijpp/riab062)
Supplement: riab062_suppl_Supplementary_Table_5 [file riab062_suppl_supplementary_table_5.docx]

*Table 5 How community pharmacy teams felt they were prepared to deal with the COVID-19 pandemic.*

| *Preparedness for the pandemic* | *n (%)* | *Participant quotes* |
| --- | --- | --- |
| *Well prepared* | 106 (17%) | **P031:** *“We got ahead of the curve our staff agreed to isolate within their families to protect the team. I*  *am extremely fortunate to have such a great team and with team work and local links feel we supported the staff and community”* [Pharmacist] |
| *Not well prepared* | 247 (38%) | **P491:** “*No, but we adapted very quickly- within 24 hours of receiving new information we had adapted.”* [Pharmacist] |
| *Somewhat prepared* | 264 (42%) | **P519:** “*The nature of the pandemic made it difficult for the NHS to prepare for or have the resources to*  *overcome the sudden increase in cases during the initial phase.*” [Pre-reg] |
| *Unsure* | 18 (3%) | **P733:** “*More so than others.*” [Healthcare assistant] |
